# The Origin and Early Evolution of Life: Prebiotic Chemistry

**DOI:** 10.3390/life9030073

**Published:** 2019-09-12

**Authors:** Michele Fiore

**Affiliations:** Université de Lyon, Claude Bernard Lyon 1, Institut de Chimie et Biochimie Moléculaires et Supramoléculaires, Batiment Lederer, Bureau 11.002, 1 Rue Victor Grignard, F–69622 Villeurbanne CEDEX, France; michele.fiore@univ-lyon1.fr; Tel.: +33-(0)472-448-080

Microfossil evidence indicates that cellular life on Earth emerged during the Paleoarchean era be-tween 3.6 and 3.2 thousand million years ago (Gya) [1]. But what is really what we call life? How, where, and when did life arise on our planet? These questions have remained most-fascinating over the last hundred years. The German biologist Carl Richard Woese emphasized the urgency of conducting in-depth studies in search of what in the early days of the formation of the universe and then of our planet, gave rise to what is called Life and he wrote “*Biology today is no more fully understood in principle than physics was a century or so ago. In both cases the guiding vision has (or had) reached its end, and in both, a new, deeper, more invigorating representation of reality is (or was) called for*.” [2] From the beginning of the last century, and in accord with what David Deamer highlighted “Life can emerge where physics and chemistry intersect” and for this reason the study of the origin of Life intersect not only the organic and inorganic chemistry but also biology, astrophysics, geochemistry, geophysics, planetology, earth science, bioinformatics, complexity theory, mathematics and philosophy from the equation. From an evolutionary chemical point of view, is possible to presume that life emerged from a mixture of inanimate matter: Organic and inorganic compounds. Such compounds reacted under favorable conditions, forming molecules that are commonly called “biotic” and that, thanks to a kind of self-organization, gave rise to the first biopolymers and to proto-metabolisms. The geology and the chemistry of Earth before the advent of life was completely different from what we know today. At that time, sunlight, volcanic heat, and hydrothermal sites were the main energy sources that could drive the synthesis of many molecules, including nucleosides, peptides, sugars and amphiphilic compounds. The atmosphere was mostly nitrogen (N_2_), as today, with a substantial amount of carbon dioxide (CO_2_) and much smaller amounts of carbon monoxide, ammonia, and methane (CO, NH_3_, CH_4_). It is also likely that water, present in locally limited amounts, contained hydrogen cyanide (HCN), formaldehyde (HCHO) and formamide (HCONH_2_). Intriguingly, those molecules are found in the interstellar space together with many other that can be considered as building blocks for the assembling of biomolecules such as water (H_2_O), formic acid (HCOOH), methanol (CH_3_OH) cyanamide (NH_2_CN), acetic acid (CH_3_COOH), acetamide (CH_3_CONH_2_), ethylene glycol (HOCH_2_CH_2_OH) and glycine [3,4]. Prebiotic chemistry experiences showed that the chemical combinations of different building blocks can give rise to the formations of different classes of biotic molecules such as 2’,3’-cyclic pyrimidine nucleotides, various–amino acids and glycerol phosphate [5,6,7,8,9,10,11]. The plausible scenarios for the assembling of these building blocks thus of such complex biomolecules are depicted as two: Hydrothermal vents and hydrothermal pools. Hydrothermal vents are systems whose heat source is the underlying magma or hot water generated by convection currents due to high thermal gradients [12]. The alternatives to hydrothermal vents are hydrothermal fields known also as hydrothermal pools. Recently, Damer and Deamer pointed out that fluctuating hydrothermal pools (FHPs) could be considered as plausibly prebiotic reactors for the synthesis of several key molecules for the development of life, including lipids, nucleic acids and peptides [13]. This short résumé is to say that the seventeen papers published in this special issue perfectly matches with the aim of the study of the origin of Life from a system chemistry and prebiotic chemistry perspective. We expect that this collection of original articles and reviews will provide the reader with an updated view of some important aspects of prebiotic chemistry thought. We hope that in the further investigations on the origin of Life will bring scientist to combine prebiotic chemistry and system chemistry in order to develop new strategies for the best understanding of how life emerged on planet based on the use of protocells models that can encapsulate sort of primitive metabolisms [14,15].
